# CIP2A is a candidate therapeutic target in clinically challenging prostate cancer cell populations

**DOI:** 10.18632/oncotarget.3875

**Published:** 2015-04-19

**Authors:** Anchit Khanna, Jayant K. Rane, Kati K. Kivinummi, Alfonso Urbanucci, Merja A. Helenius, Teemu T. Tolonen, Outi R. Saramäki, Leena Latonen, Visa Manni, John E. Pimanda, Norman J. Maitland, Jukka Westermarck, Tapio Visakorpi

**Affiliations:** ^1^ Prostate Cancer Research Center (PCRC), Institute of Biosciences and Medical Technology (BioMediTech), University of Tampere and Tampere University Hospital, Tampere, Finland; ^2^ Adult Cancer Program, The Prince of Wales Clinical School, Lowy Cancer Research Centre, UNSW Medicine, University of New South Wales, Sydney, Australia; ^3^ YCR Cancer Research Unit, Department of Biology, The University of York, Heslington, United Kingdom; ^4^ Department of Signal Processing, Tampere University of Technology, Tampere, Finland; ^5^ Centre for Molecular Medicine Norway (NCMM), Nordic EMBL Partnership, University of Oslo and Oslo University Hospital, Oslo, Norway; ^6^ Department of Cancer Prevention, Institute for Cancer Research, Oslo University Hospital, Oslo, Norway; ^7^ Turku Centre for Biotechnology, University of Turku and Åbo Akademi University, Turku, Finland; ^8^ Department of Pathology, University of Turku, Turku, Finland

**Keywords:** CIP2A, androgen receptor, castration-resistant prostate cancer, cancer stem-like cells

## Abstract

Residual androgen receptor (AR)-signaling and presence of cancer stem-like cells (SCs) are the two emerging paradigms for clinically challenging castration-resistant prostate cancer (CRPC). Therefore, identification of AR-target proteins that are also overexpressed in the cancer SC population would be an attractive therapeutic approach.

Our analysis of over three hundred clinical samples and patient-derived prostate epithelial cultures (PPECs), revealed Cancerous inhibitor of protein phosphatase 2A (CIP2A) as one such target. CIP2A is significantly overexpressed in both hormone-naïve prostate cancer (HN-PC) and CRPC patients. CIP2A is also overexpressed, by 3- and 30-fold, in HN-PC and CRPC SCs respectively. *In vivo* binding of the AR to the intronic region of CIP2A and its functionality in the AR-moderate and AR-high expressing LNCaP cell-model systems is also demonstrated. Further, we show that AR positively regulates CIP2A expression, both at the mRNA and protein level. Finally, CIP2A depletion reduced cell viability and colony forming efficiency of AR-independent PPECs as well as AR-responsive LNCaP cells, in which anchorage-independent growth is also impaired.

These findings identify CIP2A as a common denominator for AR-signaling and cancer SC functionality, highlighting its potential therapeutic significance in the most clinically challenging prostate pathology: castration-resistant prostate cancer.

## INTRODUCTION

Since AR is instrumental in the progression of prostate cancer (PC) [1], it has been the main focus of therapeutics. Androgen ablation, either chemical or surgical, has been the mainstay of treatment for advanced PC. However due to subsequent resistance to androgen ablation, this approach fails to achieve a cure. The resulting recurrent tumors are commonly known as castration-resistant prostate cancers (CRPCs). It has now been demonstrated that these cells remain androgen sensitive [2]. One third of the CRPCs harbor amplification of the AR gene [1]. In addition, rearrangements of AR, resulting in ligand independent activation of the receptor has been reported [2]. Also, in antiandrogen treated tumors, point mutations abrogating the effect of the drugs have been identified [3] and it has been shown that CRPC cells can produce low levels of androgens endogenously [4]. These adaptations make CRPCs responsive to the second line AR-signaling blocking drugs, such as abiraterone and enzalutamide. But even these second line drugs are effective only in a proportion of patients and fail to completely cure responsive patients [5]. On the other hand, the existence of cancer stem cells (SCs) in advanced stage PC and CRPCs has been suggested as another potential mechanism for resistance to drugs [6]. Several recent studies have suggested that cancer SCs are androgen-independent [7, 8]. Together these results indicate that efficient therapy for advanced stage PC would require simultaneous targeting of two types of cancer cell populations: those that are dependent on AR-signaling and the AR-independent (cells that are non-responsive to androgens) cancer stem-like cells.

CIP2A, an endogenous inhibitor of protein phosphatase 2A (PP2A), is a recently identified oncogene, which is overexpressed in several tumour types at a very high frequency [9-14]. In fact, its overexpression is emerging as one of the most frequent alterations in human cancers [13]. MYC, E2F1, AKT and mTOR have so far been identified as downstream oncogenic effectors of CIP2A [13, 15]. The clinical relevance of CIP2A, in the form of a prognostic marker, has been established for different human cancers [13]. Notably, in a study involving small number of cases, CIP2A overexpression was observed in hormone-naïve prostate cancer (HN-PC) specimens and its expression was associated with poorly differentiated and high-risk PCs [16]. However, its functional role in prostate cancer cell biology still remains elusive.

## RESULTS

**Figure 1:**
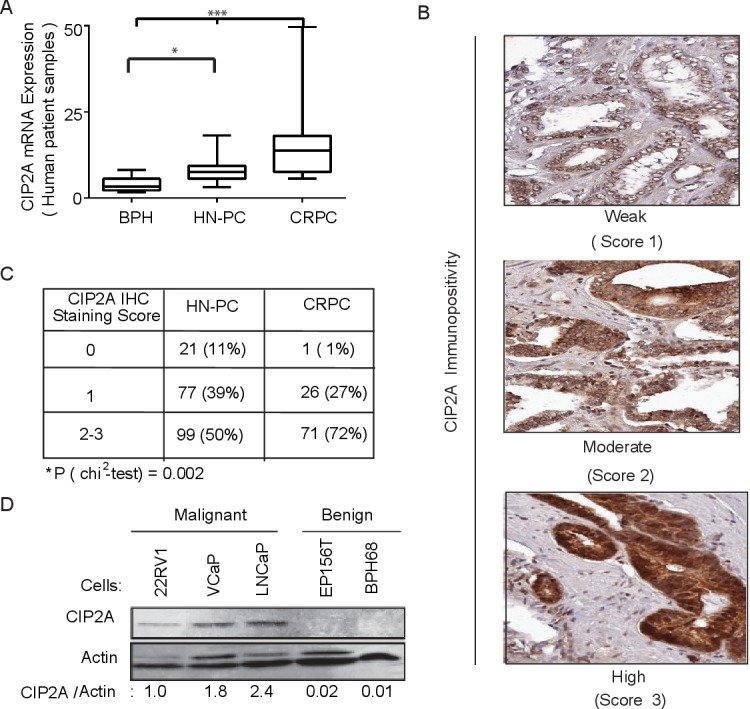
CIP2A is overexpressed in prostate cancer **A.** Overexpression of CIP2A mRNA in castration resistant prostate cancer (CRPC; *n* = 15) hormone-naïve primary prostate cancer (HN-PC; *n* = 27) compared to benign prostatic hyperplasia (BPH; *n* = 8). * = *n* < 0.05, *** = *p* value < 0.001 using the Kruskal-Wallis test with Dunn's multiple comparison post-test. **B.** CIP2A protein expression in prostate cancer specimens as detected by immunohistochemistry. A score of 0 (negative), 1 (weak), 2 (moderate) and 3 (high) was given based on the cytoplasmic CIP2A immunopositivity. **C.** Statistical analysis of CIP2A immunopositivity in both HN-PC and CRPC cases. **D.** Western blot showing CIP2A protein levels in benign and malignant prostate cancer (PC) cells.

To study the role of CIP2A in prostate cancer, we first investigated the expression and androgen regulation of this gene in clinical tissue samples, prostate cancer cell lines and different cell populations from benign prostate hypertrophy (BPH), HN-PC and CRPC patient samples. CIP2A was overexpressed in HN-PC and even more in CRPC compared to BPH at the mRNA level (Figure 1A). Also immunostaining showed higher protein levels in CRPC compared to HN-PC (Figure 1B, 1C). Notably, 99% of CRPC patient samples were positive for CIP2A expression (Figure 1C). CIP2A protein expression was also demonstrated in AR-positive PC cell lines (Figure 1D).

**Figure 2:**
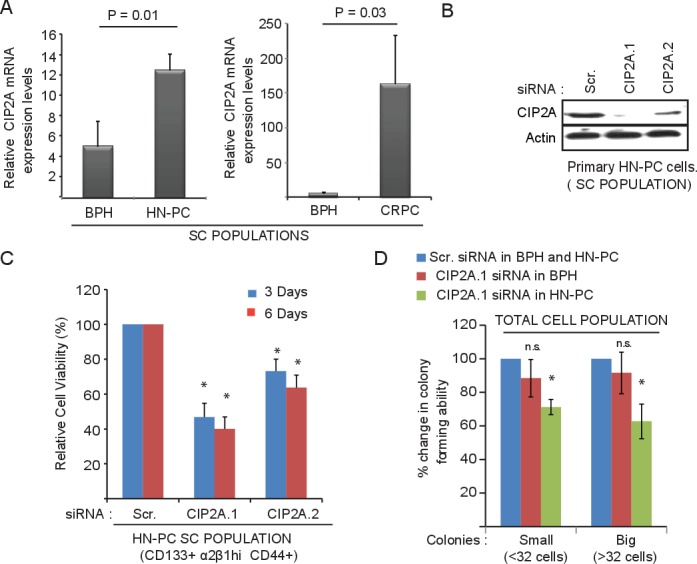
CIP2A is overexpressed and promotes growth and viability of cancer stem-like cell (SC) population from patient-derived prostate epithelial cell (PPECs) cultures **A.** Overexpression of CIP2A mRNA in the stem-like cell (SC) population of both primary HN-PC and CRPC patient samples in comparison to BPH samples. Note the y-axis scale change between HN-PC and CRPC panels. **B.** Western blot showing the effect of two different CIP2A siRNAs on CIP2A protein levels in primary HN-PC-SC cells in comparison to the Scrambled (Scr.) or control transfected cells. **C.** Effect of CIP2A depletion, using two different siRNAs against CIP2A, on cell viability of HN-PC-SC cells from patients in comparison to the control/Scr. transfected cells at 3 and 6 day time point. (* = *p*-value < 0.05 using student *t*-test) **D.** Effect of CIP2A depletion on colony forming capacity of primary cells from BPH and HN-PC patients (total cell populations) in comparison to the control/Scr. transfected cells expressed as 100% for each. Shown are the effects on both small (less than 32 cells) and big colonies (more than 32 cells ; * = *p*-value < 0.05 using student *t*-test).

Next, we assessed the expression of CIP2A in the different populations of cells from biopsy samples of BPH, HN-PC and CRPC patients. We isolated the stem-like cell (SC), transient amplifying cell (TA) and committed basal cell (CB) populations using CD133, CD44 and α_2_β_1_ cell surface markers as shown in Figure S1A. We have previously demonstrated that primary HN-PC tissue and SC cells form serially transplantable subcutaneous tumors in Rag2^−/−^γC^−/−^ immune compromised mice, which can form distant metastasis and multiple intraprostatic tumors in nude mice when grafted orthotopically in a matrigel plug containing human prostatic stroma [17]. As shown in Figure 2A, the expression of CIP2A in the cancer stem-like cell (SC) population, was increased by about three-fold in HN-PC-SCs and by about thirty-fold in CRPC-SCs compared to BPH-SCs. In contrast, CIP2A expression was decreased in TA and CB cell populations in cancer (Figure S1B, S1C). Functionally, depletion of CIP2A in the primary HN-PC PPECs cells with two different siRNAs (Figure 2B) resulted in decrease in both cell viability (Figure 2C) and colony forming capacity (Figure 2D). Particularly, the colony forming capacity in the BPH PPECs was not significantly altered on CIP2A depletion (Figure 2D). Together these results indicate that CIP2A is important for the survival of HN-PC stem-like cell (SC) populations.

**Figure 3:**
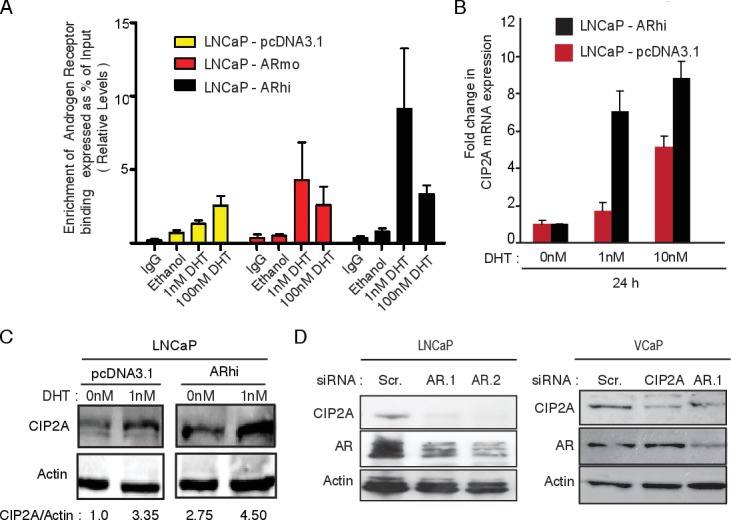
Androgen receptor (AR) binds to CIP2A and its activity and expression positively regulates CIP2A expression in prostate cancer **A.** AR binding on CIP2A intronic region. ChIP-qPCR on LNCaP-pcDNA3.1, -ARmo, -ARhi cells, hormone starved for 4 days followed by treatment with 1nM and 100nM DHT for 2h or Ethanol was performed to assess the AR recruitment on the CIP2A intronic region. **B.** Effect on CIP2A mRNA expression in LNCaP-pcDNA3.1, -ARhi cells on DHT (1nM, 10nM, 100nM) stimulation at 24h time point. **C.** Effect on CIP2A protein expression in LNCaP-pcDNA3.1, -ARhi cells on DHT (1nM) stimulation at 24h time point according to Western blotting. **D.** Effect of two independent AR siRNAs on CIP2A and AR protein expression in LNCaP cells 72h post-transfection and effect of AR and CIP2A siRNAs on CIP2A and AR protein expression in VCaP cells 72h post-transfection.

Having confirmed functional significance, we next investigated the regulation of CIP2A expression. Although, overexpression and a functional significance of CIP2A in androgen-independent cancer SCs implied AR-independent effects of CIP2A, we also have strong evidence for AR-mediated CIP2A regulation in AR-responsive cells. A statistically significant association of CIP2A immunopositivity with AR nuclear staining was observed in primary HN-PC samples (Figure S2A;[18]). ChIP-Seq data in prostate cancer cell lines from previously published studies revealed an AR binding site in CIP2A intronic region (Figure S2B). The *in vivo* functionality of this site was demonstrated by an increase in AR binding at this locus upon dihydrotestosterone (DHT) induction in LNCaP cells using ChIP-qPCR (Figure 3A). The LNCaP cells modified to expressed 2-4 (ARmo) and 6- (ARhi) fold higher levels of AR compared to control (pcDNA3.1) LNCaP cells [19] showed stronger binding (Figure 3A) upon DHT treatment. In addition, an increase in CIP2A mRNA expression was seen in LNCaP-ARhi compared to LNCaP-pcDNA3.1 cells after treatment with even modest doses of DHT (Figure 3B and S3A). Similarly, higher CIP2A protein levels were observed in LNCaP-ARhi cells in comparison to LNCaP-pcDNA3.1 cells with low DHT stimulation (Figure 3C). Also, immunofluorescene staining demonstrated higher CIP2A protein levels in LNCaP-ARhi compared to LNCaP-pcDNA3.1 cells after 1nM DHT (24h) treatment (Figure S3B). These findings are in line with our previous observations that androgen-regulated genes are induced in cells overexpressing AR, even in lower ligand concentrations [19]. Next, an independent siRNA based approach to verify the role of androgen receptor in CIP2A regulation was used. We transfected two known AR-positive prostate cancer cell lines, LNCaP and VCaP (Figure 3D), with siRNAs against AR and estimated the CIP2A protein levels. As shown in Figure 3D, AR depletion resulted in decreased CIP2A protein expression in both the AR-positive prostate cancer cell lines.

**Figure 4:**
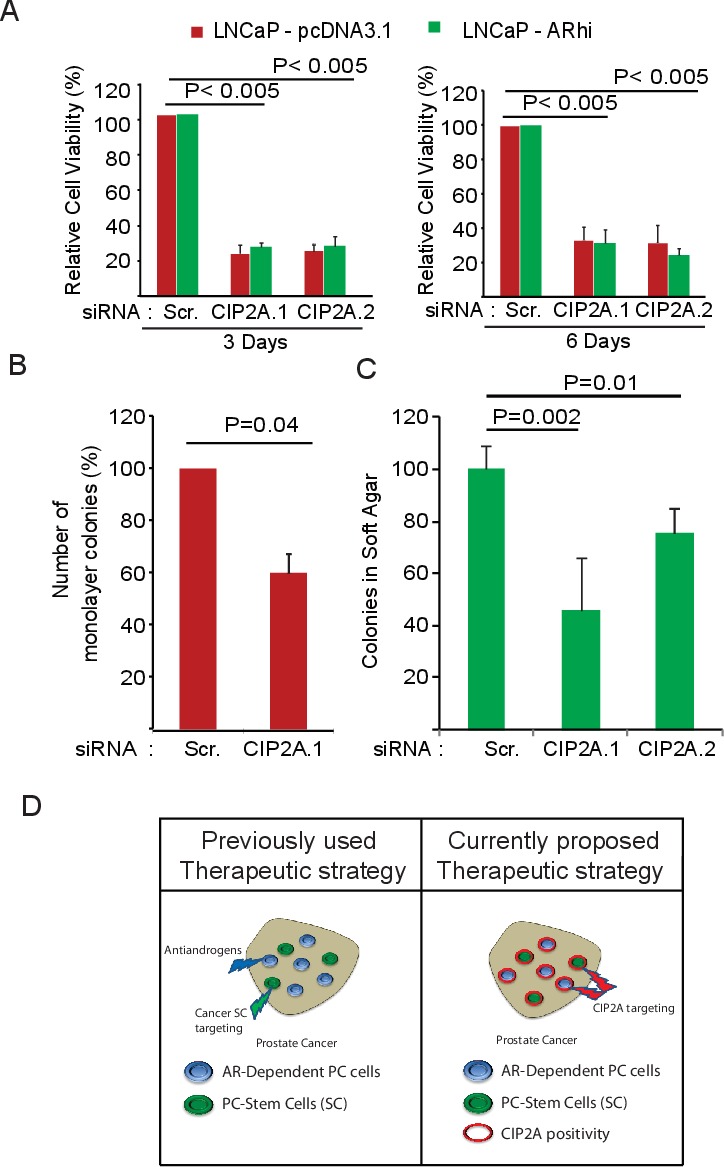
CIP2A promotes viability, clonogenicity and transformation potential of AR-positive prostate cancer cell lines **A.** Effect of two independent CIP2A siRNAs on cell viability of LNCaP-pcDNA3.1, -ARhi cells 3 and 6 days post-transfection. Student's *t*-test was used to obtain the *p* values. **B.** Effect of CIP2A siRNA on clonogenicity of LNCaP cells 6 days post-transfection. Student's *t*-test was used to obtain the *p* values. **C.** Effect of two different CIP2A siRNAs on anchorage-independent growth. Here, LNCaP cells were transfected with CIP2A.1, CIP2A.2 or Scr. siRNA and, at 48 hours post transfection, plated at low density in soft agar medium. Student's *t*-test was used to obtain the *p* values. **D.** Schematic representation of the therapeutic potential of targeting CIP2A. Since CIP2A positivity is common to both AR-dependent and cancer SC populations of prostate cancer, it surfaces as an attractive therapeutic target, especially in clinically challenging castration-resistant prostate cancers.

To assess whether the AR-mediated positive regulation of CIP2A expression has any functional consequence, we transfected LNCaP-pcDNA3.1 and LNCaP-ARhi cells with CIP2A siRNAs and performed the functional assays. As demonstrated in Figure 4A, about 4 to 5 fold decrease in cell viability was observed in both LNCaP-pcDNA3.1 and LNCaP-ARhi cells. Further, we demonstrated that CIP2A promotes clonogenicity (Figure 4B) and cellular transformation potential (Figure 4C) of LNCaP-pcDNA3.1 and -ARhi cells using monolayer clonogenic and anchorage-independent soft agar assays, respectively. These functional studies clearly identify AR as an upstream regulator of CIP2A, which in turn positively modulates cell survival.

## DISCUSSION

Overexpression of AR has been commonly observed not only in primary PCs but also in CRPC cases [1, 2, 4]. Its role in sensitizing tumor cells to low levels of androgens has been a critical deterrent for effective therapies in prostate cancer. Additionally, presence of cancer stem cells adds another layer of complexity for effective treatment, especially in CRPC cases [6]. Therefore, new therapeutic approaches that overcome these hurdles are urgently required for an effective long-term therapy against prostate cancer. This can be achieved by either using drug combinations that target AR-signaling and cancer SC-signaling simultaneously, or by identifying and targeting effector proteins common to these signaling cascades.

In this study using patient-derived prostate epithelial cultures (PPECs) and over three hundred clinical samples, we identify CIP2A to be a common denominator for AR and cancer SC-signaling functionality. We demonstrate that CIP2A is overexpressed in PC and CRPC cases and promotes the viability and clonogenicity of AR-responsive prostate cancer cells. Notably, the difference in growth in the LNCaP-pcDNA3.1 and LNCaP-ARhi cells was not clearly apparent while doing the siRNA based functional assays (Figure 4A-4C) despite different AR levels in these two populations. This could be either be due to blunting of the induction of CIP2A protein levels in ARhi cells compared to the parental LNCaP cells (1.6 fold V's 3.4 fold) as shown in Figure 3C, or due to usage of normal serum medium to carry out the knock-down assays as the stress on the cells, due to the combination of transfection reagent and presence of stripped medium or low DHT conditions, is toxic for the cells. Further, we highlighted the role of CIP2A protein in promoting the survival of the PC stem-like cell (SC) populations. Importantly, several recent studies have demonstrated the tumor suppressive role of PP2A complex in prostate cancer [20-22]. Based on these findings, we suggest therapeutic targeting of CIP2A, an established endogenous inhibitor of PP2A [10, 11, 14] as a novel approach to simultaneous target both the luminal type mass of prostate cancer, as well as the tumor initiating capacity of the SC populations (Figure 4D). However, additional mouse pre-clinical studies are required to further strengthen CIP2A's role in therapy against prostate cancer.

Altogether, as CIP2A depletion in many other cancer models have been demonstrated to result in robust antitumor effects [13, 23] and inhibition of activity of several oncogenic driver pathways [13, 15, 23], these novel results highlight the potential remedial role of CIP2A in the more resistant and therapeutically challenging prostate cancer cases.

## MATERIALS AND METHODS

### Clinical samples for mRNA expression

Freshly frozen primary prostate cancer samples from prostatectomies (8 BPH and 27 untreated or HNPC in addition to 15 CRPC specimens from transurethral resection of the prostate-treated patients) were used in the study. The samples were snap frozen in liquid nitrogen and total RNA was isolated with Trizol-Reagent (Invitrogen Inc.) according to the manufacturer's instructions. Tumor samples contained, at least, 70% cancer cells. The use of the clinical material has been approved by the ethical committee of the Tampere University Hospital.

### Tissue microarrays (TMAs)

Tissue microarrays contained 185 formalin-fixed paraffin-embedded prostatectomy and 92 CRPC (transurethral resection of the prostate) specimens obtained from Tampere University Hospital. For the prostatectomy-treated patients, detectable PSA values ≥0.5 ng/ml) in two consecutive measurements or the emergence of metastasis were considered as signs of progression. The use of tissue microarrays has been approved by the ethical committee of Tampere University Hospital and the National Authority for Medicolegal Affairs. CIP2A immunopositivity was graded in one to three tumour cores for each patient based on the intensity of the cytoplasmic immunoreactivity in the cancer cells, that is, 3 was strong, 2 moderate, 1 weak, and 0 negative. All samples were scored by, an experienced uropathologist (T. Tolonen), without knowledge of clinical status and outcome data.

### Establishment of primary prostate epithelial cultures and enrichment of prostate epithelial sub-populations

The epithelial cultures from primary prostate samples were established according to an established protocol [24]. The postoperative prostate samples were obtained with full ethical consent and were chopped into fine pieces, which were then subjected to overnight collagenase (Lorne Laboratories) treatment at 37°C. Then, the stroma was separated by differential centrifugation and prostate epithelial acini were further disrupted by mechanical and enzymatic separation methods. At this point, CD24 positive luminal cells were removed and enriched by employing CD24 MACS immunomagnetic beads (Miltenyi Biotec). The rest of the prostate epithelial cells were then grown with irradiated STOs on collagen I coated plastic plates in a stem cell media at 37°C with 5% CO_2_. Cells were sub-cultured typically when they were 80-90% confluent by splitting them into 1:3 or 1:4 proportions. At passage two; cultured epithelial cells were selected on the basis of expression of α_2_β_1_-integrin, CD44, and CD133 expression by employing rapid collagen adhesion and CD133 MACS fractionation (Figure S1A).

### Cell lines and cell culture

LNCaP cells were purchased from American Type Culture Collection (ATCC) and well established LNCaP-pcDNA3.1, LNCaP-ARmo and LNCaP-ARhi sub cell lines [19] expressing low (endogenous AR levels), moderate (two to four fold) and high AR (four to six fold) AR expression (26) respectively were also grown as per the recommended conditions. VCaP cells used were a kind gift by Dr. Jack Schalken- Radboud University, Nijmegen Medical Center, and were grown under the recommended conditions. With respect to primary cells prostate cancer directed core biopsies from three HN-PC patients had Gleason score of 4+3 while CRPCs were Gleason 9 (4+5, one sample) and 10 (5+5, 2 samples) were processed to establish cultures in stem cell medium (SCM) as described before [24].

### Quantitative (q) RT-PCR

Cells were collected from dishes and their total RNA was extracted using Trizol (Invitrogen) according to the manufacturer's instructions. First-strand cDNA synthesis was carried out from total RNA using AMV reverse transcriptase (Finnzymes) according to the manufacturer's instructions. The primers for Q-RT-PCR were designed with Primer3 program. SYBR Green II-Fast Start kit (Roche Diagnostics) and Light Cycler apparatus (Roche Diagnostics) were used for Q-RT-PCR. *TBP* (TATA box binding protein) and Actin mRNA was used as a reference. The specificity of the reactions was confirmed, in addition to the melting curve analysis, with 1.5% agarose gel electrophoresis.

Actin F- 5′- ACTTCACATCACAGCTCCCC -3′
Actin R- 5′- GAATATAATCCCAAGCGGTTTG -3′
TBP – F - 5′- CATAGGAATCCTTCTGACCCATG-3′
TBP R- 5′- CGAGCACAGAGCCTCGCCTTTGC-3′
CIP2A F- 5′-CTGGTGAGATAATCAGCAATTT-3′
CIP2A R- 5′-CGAAACATTCATCAGACTTTTCA-3′

### Small interfering RNA (siRNA) transfections

The siRNAs to inhibit CIP2A expression were obtained from Eurofins MWG operon (Ebersberg, Germany). The following double-stranded oligonucleotides were used:

CIP2A.1-5′-CUGUGGUUGUGUUUGCACUTT-3′
CIP2A.2-5′-ACCAUUGAUAUCCUU AGAATT-3′
AR.1(sense)-CCCAAGAUCCUUUCUGGGA+UU
AR.2 (sense)-GUGGAAGAUUCAGCCAAG C+UU
Scrambled-5′-UAACAAUGAGAGCACGGCTT-3′

Cells at 60%confluency were transfected with 100nM in 3 mL of antibiotic free medium (RPMI-1640 medium supplemented with 10% FCS) in each well with the respective siRNAs using Lipofectamine 2000 Reagent (Life Technologies, Carlsbad, CA), according to the manufacturer's instructions. For the primary PC cells (patient-derived prostate cancer cells, Gl-4+3), Cells at 70% confluency were transfected with 100nM of respective siRNA in 500 μL of Opti-MEM serum free medium (Life Technology) in each of the wells of a 6-well plate using Oligofectamine (Life Technology). 2mL of SCM was added after 4 hours in each well. After 4 more hours, wells were washed with PBS twice and 2 mL of fresh SCM was added. Cells were harvested at 72 hours after transfection to determine the effect on protein expression.

### Immunoflourescence

LNCaP-pcDNA3.1 and LNCaP-ARhi cells were seeded on sterilized coverslips placed in each well of a 6-well plate overnight and the cells were then treated with 1nM DHT for 24hours. Subsequently the cover slips were placed in fresh sterilized wells and then washed with PBS before being fixed using 4% paraformaldehyde for 10 min at 37° C. Then after washing with PBS twice, cells were treated with 0.5% NP-40 for 5min at room temperature. This was followed by blocking the cells with 0.5% bovine serum albumin for 30 min. Cells were then exposed to the following primary antibody dilutions, CIP2A; 1: 250 (Santa Cruz Biotechnology, Santa Cruz, CA). After which the cells were washed with PBS 3 times and incubated in FITC-conjugated goat anti Rabbit IgG (Invitrogen–Molecular Probes, Eugene, Oregon, USA) for 1h at 37°C. Next cells were then stained with the Vectashield antifade solution (Vector Laboratories) containing 0.001 mol/L 4′,6-diamidino-2- phenylindole as counterstain and cells observed under the microscope and the images taken.

### Chromatin immunoprecipitation (ChIP)

CIP2A ARBS was identified in previously published ChIP-seq dataset and ChIP was carried out as described before [25]. Chromatin was immunoprecipitated with 10 μg of normal rabbit IgG, 10 μl of anti-AR polyclonal antibody (AR3). Primer sequences used for the CIP2A ARBS amplified via qPCR examined in this work are –

CIP2A Forward -5′ –TTGTTCCTGGTCTTCCCTGT-3′,

CIP2A Reverse -5′ –GCAAAGTTTGATTGGTTTTTCC-3′

### Immunohistochemistry

Mouse Anti-CIP2A (Novus Biosciences.) was used with Power Vision+ Poly-HRP IHC kit (ImmunoVision Technologies Co., Burlingame, CA, USA) according to the manufacturer's instructions. The protocol has previously been described [18, 26].

### Western blotting

Proteins were extracted in hot Laemmli sample buffer and subjected to Western blot analysis. 30 μg total protein extracts were separated by 12% SDS-PAGE and transferred to nitrocellulose membranes. Membranes were blocked with 5% non-fat milk in TBS-0.1%-NP40 and then incubated with mouse monoclonal AR (AR441, NeoMarkers-Lab Vision Corporation, Fremont, CA, USA), goat polyclonal anti-β-Actin (Santa Cruz, Biotechnology, Santa Cruz, CA, USA) or with mouse monoclonal anti-CIP2A (Santa Cruz Biotechnology, Santa Cruz, CA). For primary PC cells CytoBuster (Merck Millipore) buffer was used to lyse freshly cultured cells to extract proteins.

### Cell viability

One day before transfection of CIP2A.1 or CIP2A.2 siRNAs, LNCaP and LNCaP-ARhi cells were seeded in RPMI-1640 medium supplemented with 10% FCS at a density of 1 × 10^3^ to 2 × 10^3^ cells per well in 96-well plates. The cells were transfected with the following-Lipofectamine 2000 reagent, 33 nM scrambled siRNA or 33 nM CIP2A.1 or CIP2A.2. Cells were then cultured for 3 and 6 days post-transfection before relative numbers of viable cells were measured by fluorescence at the 544 and 590 nm wavelengths in a FLUOstar OPTIMA Microplate Reader (BMG Labtech, Inc., Durham, NC), using the resazurin-based CellTiter-Blue Assay (Promega Corporation) according to the manufacturer's instructions. In case of primary PC cells, 20μl of cell suspension was added to an equal volume of 0.4% Trypan Blue Stain (Sigma-Aldrich). The live cells were counted using modified Neubauer's haemocytometer.

### Soft agar and monolayer clonogenic assays

At 48 hours after transfection, LNCaP (1 × 10^4^ per dish) were suspended in 1 mL of 0.25% agarose (GellyPhor; EuroClone Spa, Pero, Italy) supplemented with 2 mL complete RPMI-1640 culture medium (changed every third day). This suspension was layered over 1 mL of a base layer of 0.5% agarose in complete medium in six-well plates. After 8 or 12 days in agarose, cells were stained with Giemsa 1:20 in water (Sigma-Aldrich), the wells were scanned using the Surveyor Software (Objective Imaging Ltd.) with camera (Imaging Inc., Canada) attached to the Olympus IX71 (Olympus, Tokyo, Japan) microscope and colonies were counted by analysis with ImageJ Software (Wayne Rasband, National Institutes of Health, Betheseda, MD). Cell groupings that were greater than 1200 pixels in diameter with 3.2× enlargements were counted as colonies. In case of primary PC cells, CIP2A and scrambled siRNAs were transfected into these cells for a day, after which they were trypsynised and 250 cells were plated in each well of the 6-well Collagen-I coated plates (BD Biosciences). Cells were plated in triplicates with 2 ml of stem cell medium (SCM) and 500 μl of irradiated STO feeder cells per well. The media was changed every 2-3 days. Clonal colonies were counted at the end of 12 days under a standard light microscope. For visualization, colonies were stained with crystal violet solution (Sigma-Aldrich).

### Statistical analysis

The association between the CIP2A positivity in primary HN-PC, CRPC tumors and with AR expression was done using chi-square test. Kruskal-Wallis and student t-tests were used for other experiments. All statistical tests were two-sided.

## SUPPLEMENTARY FIGURES



## Abbreviations

AR: Androgen Receptor
CIP2A: Cancerous Inhibitor of Protein phosphatase 2A
CRPC: castration-resistant prostate cancer
PC: prostate cancer
HN-PC: hormone-naïve prostate cancer
BPH: benign prostate hypertrophy
PPECs: patient-derived prostate epithelial cultures
DHT: dihydrotestosterone
SC: stem-like cell
TA: transient amplifying cell
CB: committed basal cell
