# Supplementary material for: CIP2A is a candidate therapeutic target in clinically challenging prostate cancer cell populations
Source: Oncotarget. 2015 Apr 19;6(23):19661–70. doi: 10.18632/oncotarget.3875 (PMC4637312; doi:10.18632/oncotarget.3875)
Supplement: Supplementary file 1 [file oncotarget-06-19661-s001.pdf]

CIP2A is a candidate therapeutic target in clinically challenging prostate cancer cell populations

Supplementary Material and Methods

A

Cell sub-population	Surface Marker Profile
Stem Cells (SC)	CD133+ α2β1hi CD44+
Transit Amplifying (TA) Cells	CD133 - α2β1hi CD44+
Committed Basal (CB) Cells	CD133 - α2β1lo CD44+

B

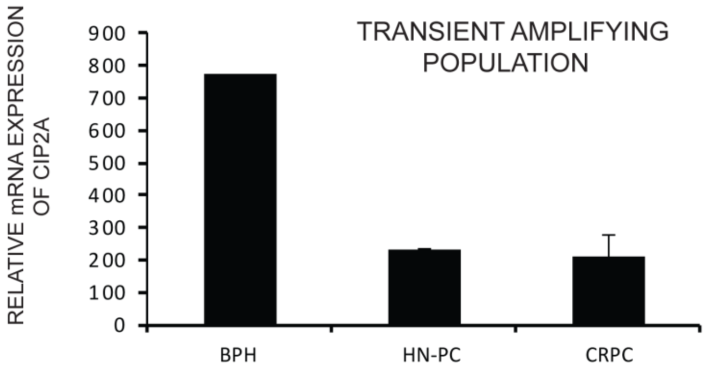

C

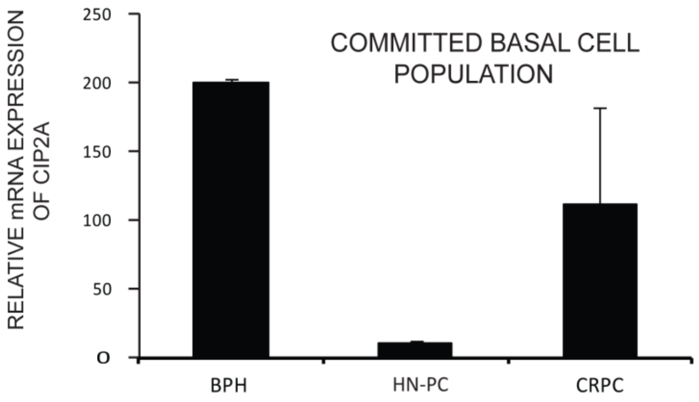

Supplementary Figure S1

A. Surface marker profile used for isolating stem-like cells (SC), transit amplifying (TA) cells and committed basal (CB) cells from patients (n=3).
B and C. Expression of CIP2A mRNA in the indicated populations of both primary hormone naive prostate cancer (HN-PC) and CRPC patient samples in comparison to BPH samples.

A

CIP2A IHC Staining Score	Androgen Receptor (AR) Staining Score	
	0-1 (negative)	2-3 (positive)
0	10	7
1	16	57
2-3	10	84

* P (chi^2-test) = 0.001

B

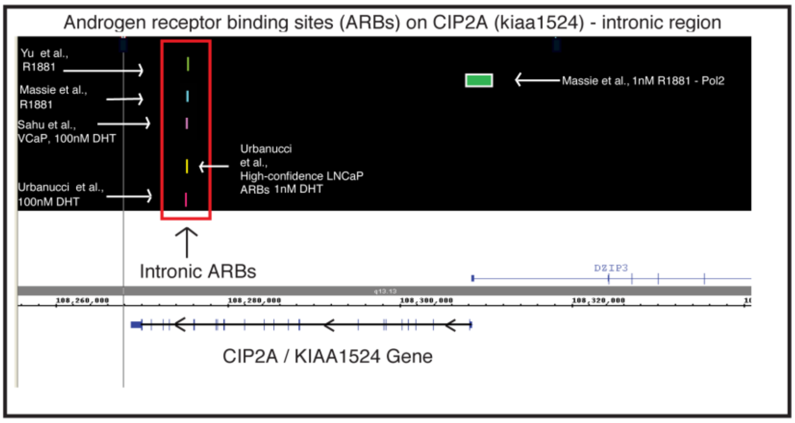

Supplementary Figure S2

A. Statistical analysis of association of CIP2A immunopositivity with AR nuclear staining in primary prostate cancer cases.

B. Androgen receptor binding sites (ARBs) in the intronic region of CIP2A as observed in indicated published studies. The promoter region of CIP2A is bound by RNA Pol2 upon androgen stimulation according to Massie et al., dataset. The promoter and the intronic region describe above are in open chromatin sites according to He et al., (DNase I hypersensitivity (DHS) data in LNCaP cells; data not shown).

References:

Yu J, Yu J, Mani RS, et al. An integrated network of androgen receptor, polycomb, and TMPRSS2-ERG gene fusions in prostate cancer progression. Cancer Cell 2010;17(5):443-54

Massie CE, Lynch A, Ramos-Montoya A, et al. The androgen receptor fuels prostate cancer by regulating central metabolism and biosynthesis. EMBO J 2011;30(13):2719-33.

Urbanucci A, Sahu B, Seppala J, et al. Overexpression of androgen receptor enhances the binding of the receptor to the chromatin in prostate cancer. Oncogene 2012;31(17):2153-63.

He HH, Meyer CA, Chen MW, et al. Differential DNase I hypersensitivity reveals factor-dependent chromatin dynamics. Genome Res. 2012 Jun;22(6):1015-25

Sahu B, Laakso M, Pihlajaama P. FoxA1 specifies unique androgen and glucocorticoid receptor binding events in prostate cancer cells. Cancer Res. 2013;1;73(5):1570-80.

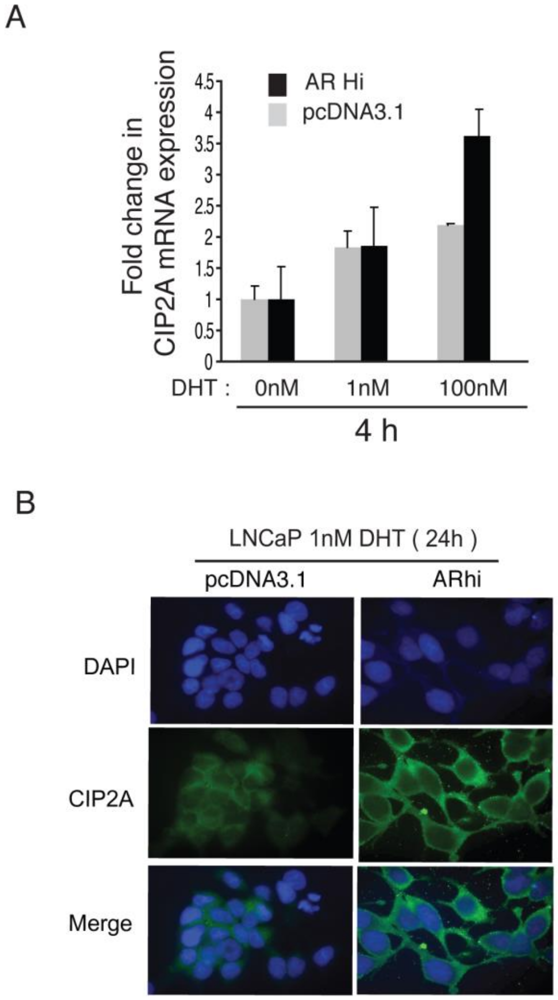

Supplementary Figure S3

A. Effect of DHT (at indicated concentrations) on CIP2A mRNA expression in LNCaP cells and ARhi LNCaP cells.

B. Comparison of CIP2A protein levels between LNCaP-pcDNA3.1 and -ARhi cells on DHT (1nM) stimulation at 24h time point according to immunofluorescence staining
